# Desvendando os Desafios no Diagnóstico da Amiloidose Cardíaca

**DOI:** 10.36660/abc.20240107

**Published:** 2024-05-07

**Authors:** Lorena Squassante Capeline

**Affiliations:** 1 Universidade Federal de São Paulo Escola Paulista de Medicina São Paulo SP Brasil Universidade Federal de São Paulo - Escola Paulista de Medicina (UNIFESP - EPM), São Paulo, SP – Brasil

**Keywords:** Amiloidose Familiar/diagnóstico por imagem, Cardiomiopatia Restritiva, Pré-albumina, Cromossomos Humanos Par 18, Diagnóstico por Imagem/métodos, Ecocardiografia/métodos, Cintilografia/métodos

A amiloidose transtirretina (ATTR) é uma patologia genética rara que leva à cardiomiopatia infiltrativa potencialmente fatal, representando a forma mais comum de cardiomiopatia restritiva hereditária.^1^ O gene da transtirretina (TTR) está localizado no cromossomo 18q12.1 com herança autossômica dominante e penetrância variável.^2^ A ATTR apresenta manifestações clínicas heterogêneas, sendo o envolvimento cardíaco o principal marcador de mau prognóstico. A amiloidose cardíaca (AC), uma condição rara e muitas vezes negligenciada, emergiu como um desafio significativo no campo da cardiologia. Esse distúrbio apresenta uma série de dificuldades para o diagnóstico precoce e preciso.^1 – 6^

Atualmente, a disponibilidade de testes genéticos identificou uma população portadora da variante genética sem o fenótipo de AC.^1 – 3^ Contudo, devido à evolução progressiva e lenta da doença, a identificação exata do envolvimento cardíaco permanece desconhecida.^2 – 4^

A complexidade da AC reside, em parte, na diversidade de sintomas que pode apresentar. Desde fadiga e falta de ar até irregularidades cardíacas, os sinais podem ser atribuídos a uma variedade de condições, tornando a identificação da AC um desafio para os cardiologistas. A AC é frequentemente confundida com outras cardiopatias mais comuns, resultando em diagnósticos tardios ou até mesmo incorretos.^1^ A busca por métodos diagnósticos mais eficazes e acessíveis é urgente.

Nesse contexto, exames diagnósticos como a ecocardiografia bidimensional com speckle-tracking (2D-STE), a ressonância magnética cardíaca ou a cintilografia miocárdica com pirofosfato têm sido a base para o rastreamento e monitoramento dessa condição.^1 – 3 , 7 – 9^ Apesar dos avanços tecnológicos, o 2D-STE continua sendo a pedra angular na avaliação inicial do envolvimento cardíaco em cardiomiopatias devido à sua não invasividade, precisão, baixo custo e facilidade de acessibilidade.^8^

No entanto, a utilidade do 2D-STE para a análise da deformação biventricular não é relatada em uma população portadora sem AC. Alguns estudos demonstraram que o strain biventricular global e regional é menor em indivíduos com TTR geneticamente confirmada antes do desenvolvimento da AC.^10 , 11^

Na busca pelo diagnóstico precoce, em “Avaliação cintilográfica e ecocardiográfica em portadores de variantes patogênicas ou provavelmente patogênicas do gene TTR sem envolvimento cardíaco manifesto”,^12^ publicado nesta edição, avaliou o envolvimento cardíaco na ATTR por meio de cintilografia com pirofosfato e ecocardiografia com strain para identificar detecção precoce de comprometimento cardíaco nesses pacientes.^12^

Para responder a essa questão, os autores realizaram um estudo transversal com uma amostra de conveniência de pacientes acompanhados no ambulatório de doenças raras de um centro terciário brasileiro. Esses pacientes tinham diagnóstico confirmado de polineuropatia amiloide familiar (PAF) ou eram parentes do caso índice da forma neurológica da doença.^12^

O objetivo foi avaliar a prevalência de envolvimento cardíaco subclínico; analisar achados de cintilografia e ecocardiografia; avaliar a associação entre a presença de PAF e envolvimento cardíaco subclínico; e determinar se uma mutação específica pode aumentar o envolvimento cardíaco. O estudo focou em uma população adulta com diagnóstico de variante genética no gene da transtirretina associada à forma neurológica, mas que era cardiovascularmente assintomática.^12^ Foram avaliados 23 pacientes, dos quais 9 (39,1%; IC 95% = 29–49%) preencheram os critérios para envolvimento cardíaco, com 6 (26%) preenchendo critérios baseados apenas no strain longitudinal global (SGL). Não houve associação entre PAF e portadores assintomáticos, avaliada por ecocardiograma de strain e cintilografia com pirofosfato (p=0,19). A velocidade da onda e’ septal foi a única variável que diferiu significativamente entre indivíduos com e sem SLG reduzido, com área sob a curva ROC de 0,80 (IC 95% = 0,61–0,98, p=0,027). A melhor acurácia diagnóstica foi alcançada com velocidade da onda e’ septal menor ou igual a 8,5 cm/s. Não houve associação entre o tipo de mutação e a presença de envolvimento cardíaco pré-clínico (37,5%, p=0,90).^12^

Na interpretação desses resultados é fundamental considerar o desenho do estudo, que representa uma amostra parcial da população de pacientes atendidos no centro terciário, o fato de a ecocardiografia ter sido realizada por um único profissional e o pequeno tamanho da amostra. Os autores reconhecem essas limitações.

Para concluir, Silva et al.,^12^ demonstraram que o envolvimento cardíaco subclínico foi frequente em uma amostra de portadores cardiovasculares assintomáticos da variante genética da TTR. O achado ecocardiográfico mais comum no estudo foi a redução do strain longitudinal global do ventrículo esquerdo. Não houve associação entre PAF e envolvimento cardíaco subclínico, e nem o tipo de variante genética foi associado ao envolvimento cardíaco precoce.

Globalmente, dado o conhecimento de que na AC, a deposição de proteínas amiloides é progressiva e as complicações cardiovasculares são inevitáveis, o diagnóstico precoce do envolvimento cardíaco pela ATTR torna-se crucial. Além disso, existe atualmente um arsenal terapêutico que aumenta a sobrevida nesta condição. Pensando nisso, como cardiologistas, devemos agir com rigor e precisão para retardar a progressão desta patologia e melhorar a qualidade de vida dos nossos pacientes.
