# Supplementary material for: Desvendando os Desafios no Diagnóstico da Amiloidose Cardíaca
Source: Arq Bras Cardiol. 2024 May 7;121(4):e20240107. [Article in Portuguese] doi: 10.36660/abc.20240107 (PMC11081089; doi:10.36660/abc.20240107)
Supplement: Translation

# Translation: Unraveling the Challenges in Diagnosing Cardiac Amyloidosis

**DOI:** 10.36660/abc.20240107i

**Authors:** Lorena Squassante Capeline

**Affiliations:** 1 São Paulo SP Brazil Universidade Federal de São Paulo - Escola Paulista de Medicina (UNIFESP - EPM), São Paulo, SP – Brazil

**Keywords:** Amyloidosis, Hereditary, Transthyretin-Related, Cardiomyopathy, Restrictive, Echocardiography

Transthyretin amyloidosis (ATTR) is a rare genetically caused pathology leading to potentially fatal infiltrative cardiomyopathy, representing the most common form of hereditary restrictive cardiomyopathy.^1^The transthyretin (TTR) gene is located on chromosome 18q12.1 with autosomal dominant inheritance and variable penetrance.^2^ATTR exhibits heterogeneous clinical manifestations, with cardiac involvement being the primary marker of poor prognosis. Cardiac amyloidosis (CA), a rare and often overlooked condition, has emerged as a significant challenge in the field of cardiology. This disorder poses a series of difficulties regarding early and accurate diagnosis.^1 – 6^

Currently, the availability of genetic testing has identified a population carrying the genetic variant without the phenotype of CA.^1 – 3^However, due to the progressive and slow evolution of the disease, the exact identification of cardiac involvement remains unknown.^2 – 4^

The complexity of CA lies, in part, in the diversity of symptoms it may present. From fatigue and shortness of breath to cardiac irregularities, the signs can be attributed to a variety of conditions, making the identification of CA a challenging puzzle for cardiologists. CA is often confused with other more common heart diseases, resulting in delayed or even incorrect diagnoses.^1^The search for more effective and accessible diagnostic methods is urgent.

In this context, diagnostic tests such as two-dimensional echocardiography with speckle-tracking (2D-STE), cardiac magnetic resonance imaging, or myocardial scintigraphy with pyrophosphate have been the foundation for screening and monitoring this condition.^1 – 3 , 7 – 9^Despite technological advancements, 2D-STE remains the cornerstone in the initial assessment of cardiac involvement in cardiomyopathies due to its non-invasiveness, accuracy, low cost, and ease of accessibility.^8^

However, the utility of 2D-STE for the analysis of biventricular strain is not reported in a carrier population without CA. Some studies have shown that global and regional biventricular strain is lower in individuals with genetically confirmed TTR before the development of CA.^10 , 11^

In the pursuit of early diagnosis, “Scintigraphic and echocardiographic evaluation in carriers of pathogenic or likely pathogenic variants of the TTR gene without manifest cardiac involvement,”^12^published in this edition, assessed cardiac involvement in ATTR through pyrophosphate scintigraphy and echocardiography with strain to identify early detection of cardiac impairment in these patients.^12^

To address this question, the authors conducted a cross-sectional study on a convenience sample of patients followed at the rare diseases outpatient clinic of a Brazilian tertiary center. These patients had a confirmed diagnosis of familial amyloid polyneuropathy (FAP) or were relatives of the index case of the neurological form of the disease.^12^

The objective was to assess the prevalence of subclinical cardiac involvement; analyze findings from scintigraphy and echocardiography; evaluate the association between the presence of FAP and subclinical cardiac involvement; and determine if a specific mutation could increase cardiac involvement. The study focused on an adult population diagnosed with a genetic variant in the transthyretin gene associated with the neurological form but who were asymptomatic cardiovascularly.^12^Twenty-three patients were evaluated, of whom 9 (39.1%; 95% CI = 29–49%) met the criteria for cardiac involvement, with 6 (26%) meeting criteria based solely on global longitudinal strain (GLS). There was no association between PAF and asymptomatic carriers, as assessed by strain echocardiography and pyrophosphate scintigraphy (p=0.19). The septal e’ wave velocity was the only variable that differed significantly between individuals with and without reduced GLS, with an area under the ROC curve of 0.80 (95% CI = 0.61–0.98, p=0.027). The best diagnostic accuracy was achieved with a septal e’ wave velocity less than or equal to 8.5 cm/s. There was no association between the type of mutation and the presence of pre-clinical cardiac involvement (37.5%, p=0.90).^12^

In interpreting these results, it is essential to consider the study's design, which represents a partial sample of the patient population treated at the tertiary center, the fact that echocardiography was performed by a single professional and the small sample size. The authors acknowledge these limitations.

In conclusion, Silva et al.^12^demonstrated that subclinical cardiac involvement was frequent in a sample of asymptomatic cardiovascular carriers of the genetic variant for TTR. The most common echocardiographic finding in the study was a reduction in the global longitudinal strain of the left ventricle. There was no association between PAF and subclinical cardiac involvement, and neither the type of genetic variant was associated with early cardiac involvement.

Overall, given the knowledge that in CA, the deposition of amyloid proteins is progressive and cardiovascular complications are inevitable, early diagnosis of cardiac involvement by ATTR becomes crucial. Furthermore, there is currently a therapeutic arsenal that increases survival in this condition. With this in mind, as cardiologists, we must act with accuracy and precision to delay the progression of this pathology and improve the quality of life for our patients.
